# A large bioassay identifies *Stb* resistance genes that provide broad resistance against Septoria tritici blotch disease in the UK

**DOI:** 10.3389/fpls.2022.1070986

**Published:** 2023-01-09

**Authors:** Henry Tidd, Jason J. Rudd, Rumiana V. Ray, Ruth Bryant, Kostya Kanyuka

**Affiliations:** ^1^ Protecting Crops and the Environment, Rothamsted Research, Harpenden, United Kingdom; ^2^ Division of Plant and Crop Sciences, School of Biosciences, University of Nottingham, Sutton Bonington, Loughborough, United Kingdom; ^3^ RAGT Seeds, Ickleton, United Kingdom; ^4^ NIAB, Cambridge, United Kingdom

**Keywords:** Zymoseptoria tritici, septoria tritici blotch, wheat, disease resistance, crop disease, bioassay

## Abstract

**Introduction:**

Septoria tritici blotch (STB) is one of the most damaging fungal diseases of wheat in Europe, largely due to the paucity of effective resistance genes against it in breeding materials. Currently dominant protection methods against this disease, e.g. fungicides and the disease resistance genes already deployed, are losing their effectiveness. Therefore, it is vital that other available disease resistance sources are identified, understood and deployed in a manner that maximises their effectiveness and durability.

**Methods:**

In this study, we assessed wheat genotypes containing nineteen known major STB resistance genes (Stb1 through to *Stb19*) or combinations thereof against a broad panel of 93 UK *Zymoseptoria tritici* isolates. Seedlings were inoculated using a cotton swab and monitored for four weeks. Four infection-related phenotypic traits were visually assessed. These were the days post infection to the development of first symptoms and pycnidia, percentage coverage of the infected leaf area with chlorosis/necrosis and percentage coverage of the infected leaf area with pycnidia.

**Results:**

The different *Stb* genes were found to vary greatly in the levels of protection they provided, with pycnidia coverage at four weeks differing significantly from susceptible controls for every tested genotype. *Stb10*, *Stb11*, *Stb12*, *Stb16q*, *Stb17*, and *Stb19* were identified as contributing broad spectrum disease resistance, and synthetic hexaploid wheat lines were identified as particularly promising sources of broadly effective STB resistances.

**Discussion:**

No single *Z. tritici* isolate was found to be virulent against all tested resistance genes. Wheat genotypes carrying multiple *Stb* genes were found to provide higher levels of resistance than expected given their historical levels of use. Furthermore, it was noted that disease resistance controlled by different *Stb* genes was associated with different levels of chlorosis, with high levels of early chlorosis in some genotypes correlated with high resistance to fungal pycnidia development, potentially suggesting the presence of multiple resistance mechanisms.

The knowledge obtained here will aid UK breeders in prioritising Stb genes for future breeding programmes, in which optimal combinations of resistance genes could be pyramided. In addition, this study identified the most interesting Stb genes for cloning and detailed functional analysis.

## Introduction

Septoria tritici blotch (STB), caused by the fungal pathogen *Zymoseptoria tritici*, is one of the most damaging wheat diseases across Europe, with the capacity to cause up to 50% crop losses under disease-favourable conditions (Fones and Gurr, 2015). Approximately 70% of the fungicides used in Europe can be for the purpose of preventing *Z. tritici* epidemics (Duveiller et al., 2007; Torriani et al., 2015). Developing methods for protecting wheat from STB is therefore a high priority for UK wheat breeders and researchers.

Traditionally, STB protection has been achieved through the widespread application of fungicides reinforced with the deployment of a small number of *Stb* resistance genes. However, the sexual reproductive cycle that *Z. tritici* undergoes around the end of the cropping season can contribute to high levels of genetic diversity in the pathogen, leading to the rapid loss of effectiveness from fungicides. Resistant strains now exist for every major fungicide group used against them (Fraaije et al., 2005; Cools and Fraaije, 2008; Stammler and Semar, 2011; Hillocks, 2012; van den Berg et al., 2013; Estep et al., 2015), or their development has been demonstrated to be possible through directed evolution, e.g. the case of quinone inside inhibitors (Fouché et al., 2021). A similar lack of durability has proven an issue with *Stb* resistance genes. For example, *Stb6* and *Stb15* have both been widely used in Northern Europe and were initially highly effective however, both have since been widely broken by *Z. tritici* due to the selection pressures caused by their widespread use (Chartrain, 2004b; Arraiano et al., 2009; Stephens et al., 2021). *Stb16q* has also been brought into wide use more recently in some European countries, and initially offered very broad STB resistance. However, isolates of *Z. tritici* virulent on wheat cultivars carrying *Stb16q* have already been reported in Iran, Ireland and France (Dalvand et al., 2018; Kildea et al., 2020; Orellana-Torrejon et al., 2022a) and are likely to spread rapidly within field populations, making this resistance gene less useful in future breeding programmes. The lack of broad spectrum STB resistance in wheat leaves agricultural systems vulnerable when major resistance genes are broken (e.g. the cultivar Gene in the USA, which was fully resistant in 1992 but become widely susceptible by 1995, causing substantial crop losses (Cowger et al., 2000), or Cougar, which has become unpopular due to the development of Cougar-virulent strains of *Z. tritici* in the UK (Kildea et al., 2021). Such problems will only become more frequent as effective fungicide protection options become more limited (Birr et al., 2021).

It is also noteworthy that some individual major resistance genes that have been widely used in breeding so far have proved to be more durable than others. For example, *Stb1* was introduced to the grower market in the cultivar Oasis in 1975 and has been used in many other cultivars (e.g. Sullivan) since 1979 and remained effective in the field up until mid-2000’s (Cowger et al., 2000; Adhikari et al., 2004b; Singh et al., 2016). *Stb4* also proved to be reasonably durable, lasting for approximately 15 years. After its introduction to breeding programs in 1975 (in a cross between Tadorna, Cleo and Inia 66), the first cultivar containing *Stb4* underwent a commercial release in 1984 (Somasco et al., 1996), and this gene remained effective until 2000 (Jackson et al., 2000). However, no individual *Stb* gene so far identified appears to be completely durable. Gene pyramiding may be able to mitigate this rapid breakdown of disease resistance by producing additional obstacles to fungal populations in the evolution of new virulences. For example, Kavkaz-K4500 is one of the most durable sources of field resistance used for breeding and has been shown to possess at least five qualitative resistance genes, including *Stb6*, *Stb10* and *Stb12* (Chartrain et al., 2005a). This combination of *Stb* genes seems to be sufficient to make Kavkaz-K4500 resistant to STB under field conditions despite the fact that many international *Z. tritici* isolates are virulent on it in laboratory tests (Chartrain et al., 2004a; Chartrain et al., 2005a) – this may suggest high genetic diversity differences between UK and international *Z. tritici* populations, or could be related to the different levels of inoculum used in laboratory vs field trials.

The currently limited availability of data on the interaction between modern *Z. tritici* isolates and wheat [due to limited numbers of isolates being tested in most studies and the fact that many older isolates are reused in many studies for example, 22 isolates from one set of plots at a single location were used in Cowger et al. (2000), ten isolates from a range of Iranian farms in Dalvand et al. (2018) and only one 1996 isolate in Ali et al. (2008)], along with the difficulty in comparing data from different sources, is problematic as it has limited our ability to identify useful sources of quantitative resistances to this disease (Chartrain et al., 2004a). This combined with the limited historical breeding for STB resistance, has led to a dearth of cultivars with significant quantitative resistance to the disease.

Further issues arise from the lack of standardised, modern wild-type *Z. tritici* isolates among the standard model strains for this disease, which represents a significant obstacle to the development of durable STB resistance in wheat due to the difficulties it causes in designing experiments that produce useful information on the likely field efficacy of resistance genes and quantitative trait loci (QTLs) for breeders and can be easily compared to other work in the same field. It is therefore important that new field isolates of *Z. tritici* are collected from all regions of interest for breeders to be used in the testing of new resistance genes. A database of *Z. tritici* isolates with known virulence profiles could help identify combinations of *Stb* resistance genes that could provide several independent resistances for each tested *Z. tritici* isolate. This could allow us to identify combinations of resistance genes that would require several independent mutations in any *Z. tritici* isolate in order for that isolate to gain virulence.

This rapid breakdown of existing resistances makes it particularly important that breeders have access to novel STB resistance genes effective against local *Z. tritici* populations. Several known major resistance genes, such as *Stb5*, *Stb17* and *Stb19*, have not previously been widely used in Europe, and could perhaps be used to replace those that have already been overcome (e.g. *Stb6* and *Stb16q*). Unfortunately, little data is currently available to breeders regarding which of these genes are sufficiently broadly effective to be worth using in breeding programs.

It is therefore clear that a future priority in wheat breeding is likely to be the development of elite lines containing a greater variety of disease resistance genes. Major resistance genes are likely to be a large part of this as they can be identified easily and applied quickly in breeding programs, and major genes not yet broken will provide excellent field resistance. More than twenty *Stb* resistance genes that could be used in wheat breeding programs have thus far been identified, providing natural protection against a variety of *Z. tritici* isolates at the different stages of the wheat life cycle (referred to as seedling and adult resistance genes) (Dreisigacker et al., 2015). For many of these *Stb* genes we have some information relating to their chromosomal locations, but in the majority of cases this data is imprecise.

Overall, large pathology screens are necessary to assess the effectiveness of *Stb* genes more accurately. Conducting these screens on more genetically diverse germplasm (particularly non-elite landraces and ancestor species) may help to identify novel *Stb* genes highly effective against current *Z. tritici* populations. Here we carried out a broad screen of 2015-2017 UK *Z. tritici* isolates against a panel of wheat lines of diverse origin containing known *Stb* resistance genes to produce estimates of the effectiveness of each of these genes against contemporary field populations of *Z. tritici* in the UK. Several *Stb* genes were identified as contributing broad spectrum disease resistance, and synthetic hexaploid wheat lines were identified as promising sources of broadly effective STB resistance.

## Materials and methods

### Library of fungal isolates

One hundred *Z. tritici* isolates were donated by Bart Fraaije (NIAB, UK). These isolates were collected from locations around the UK in the years 2015-2017. These isolates were originally drawn from many sources with different naming conventions, and were renamed for ease of use in this project – a list of the original names of these isolates on receipt is included in the **Supplementary Data**.

In preparation for use in these experiments, the isolates were grown on 7% (w/v) YPD agar (Formedium Ltd., Hunstanton, UK) plates containing 1 unit of penicillin and 1 µg/mL streptomycin (Merck Life Science UK Limited, Gillingham, UK) to remove bacterial contamination. Approximately 25 µl of original *Z. tritici* glycerol stocks were used per plate. Inoculated plates were incubated at 16°C for four to seven days before the fungus was harvested using a sterile loop into 50% (w/v) glycerol and stored at -80°C. This was then repeated using antibiotic free YPD agar plates to ensure the fungi used were not stressed. Fungi from antibiotics-free plates were harvested and stored identically.

Where bacterial contaminants proved resistant to the antibiotics used, contaminated glycerol stock was diluted (approximately by a factor of 100, depending on concentration), allowing individual colonies to form from single spores or cells. Suitable uncontaminated *Z. tritici* colonies were harvested into 50% glycerol and re-plated to produce pure stocks.

### Wheat lines used

Wheat lines were chosen for use in this study that collectively contained *Stb* resistance genes *Stb1-Stb19*. These lines and the *Stb* genes they contain are listed in **Table 1**. Taichung 29 and KWS Cashel were both included as known susceptible controls (of these, KWS Cashel was the primary control and Taichung 29 was included as a second control in case KWS Cashel was found to be resistant to any *Z. tritici* isolates used).

**Table 1:** Wheat lines used in this study with known *Stb* genes.

Wheat Genotype	Known *Stb* genes	Reference
**Taichung 29**	No *Stb* genes known	-
**KWS Cashel**	No *Stb* genes known	-
**Bulgaria 88**	*Stb1, Stb6*	Adhikari et al., 2004b
**Veranopolis**	*Stb2, Stb6*	Liu et al., 2013
**Israel 493**	*Stb3, Stb6*	Goodwin et al., 2015
**Tadinia**	*Stb4, Stb6*	Adhikari et al., 2004a
**Synthetic 6X**	*Stb5*	Arraiano et al., 2001
**Estanzuela Federal**	*Stb7*	McCartney et al., 2003
**Synthetic M6 (Previously W7984)**	*Stb8*	Adhikari et al., 2003
**Tonic**	*Stb9*	Chartrain et al., 2009
**Kavkaz-K4500**	*Stb6, Stb7, Stb10, Stb12*	Chartrain et al., 2005a
**TE9111**	*Stb6, Stb7, Stb11*	Chartrain et al., 2005b
**Salamouni**	*Stb6, Stb13, Stb14*	Cowling, 2006
**Riband**	*Stb15*	Arraiano et al., 2007
**Synthetic M3**	*Stb16q, Stb17*	Tabib Ghaffary et al., 2012
**Balance**	*(Stb6), Stb18*	Tabib Ghaffary et al., 2011
**Lorikeet**	*Stb6, Stb19*	Yang et al., 2018

### Inoculation of wheat plants

*Z. tritici* isolates used in inoculations were cultured on antibiotic-free YPD agar plates and grown for four to seven days at 16°C. Fungal blastospores were then harvested using sterile loops into 5mL of 0.1% Silwet L-77 surfactant (Momentive Performance Materials, Waterford, NY, USA) in H_2_O and diluted to a concentration of 10^7^ spores per mL using the average of two replicated measurements from a haemocytometer. High concentrations and the presence of a surfactant are not reflective of field conditions but were included to encourage rapid infection to reduce the time needed per bioassay.

Plants were grown for approximately three weeks (adapted for variable growth rates where necessary) at 16-hour day, 8-hour night cycles under halogen or white LED lamps at a temperature of 21°C and ambient humidity. After inoculation, these plants were transferred to 17°C and the same 16-hour day, 8-hour night cycle. The second leaf was inoculated where possible, although for some cultivars (e.g. Israel 493) the third leaves were used due to their larger size. One leaf each from a minimum of three plants was used for testing each wheat genotype - *Z. tritici* isolate interaction.

Leaves were affixed to aluminium inoculation tables using double sided sticky tape and rubber bands, which also defined the area inoculated and scored. Cotton buds were used to inoculate each spore suspension onto leaves of three plants of each wheat line (four strokes per leaf, ensuring an even layer of moisture on leaf surface). Non-inoculated leaves were trimmed to ensure light access to inoculated leaves.

After inoculation, plants were placed in high humidity boxes (**Supplementary Figure 1**) for three days before the inner tray (perforated to allow for water uptake) was removed and placed in a larger plastic watering tray to minimise the risk of causing leaf damage or cross-contamination from direct watering.

Plants were maintained for 28 days after inoculation to allow symptom development. They were watered three times per week and kept trimmed to ensure light access to inoculated leaves. From ten days post inoculation (dpi), plants were checked regularly (every two days where possible) for chlorosis, necrosis and pycnidia development, and symptoms were recorded. Photographs were taken at each check for later verification.

The final screen included 973 tested interactions. Due to the large number of wheat genotype – *Z. tritici* isolate interactions tested, one replicate was normally performed for each of these interactions in the bioassay.

### Visual symptom assessments

Necrosis, chlorosis and pycnidia development symptoms were assessed visually. Assessment of the rate of symptom and pycnidia development began ten days after seedling inoculation by *Z. tritici* for each plant. Assessments were then carried out three times a week at regular intervals until 28 days after the initial inoculation date. Leaf status was recorded as no infection (i.e. clean), chlorosis present (showing yellow chlorotic tissue but which had not yet progressed to necrosis), necrosis present (where necrotic lesions were visible), chlorosis with pycnidia (chlorotic symptoms present with small black pycnidia visible on the inoculated leaf surface) or necrosis with pycnidia. The first date on which chlorosis or necrosis was seen was used to determine the “days until symptom development” trait value, while the date on which pycnidia were first noted was used to determine the “days until pycnidia development” trait value. Photographs were taken at each check in case needed for later verification of results.

At 28 days post infection, before leaves were harvested, the “percentage leaf area covered by symptoms” and “percentage leaf area covered by pycnidia” traits were visually assessed. The values for each leaf were rounded to 0, 20, 40, 60, 80 or 100% for each leaf. Photographs were taken in case needed for later verification of results.

### Statistical analysis

Statistical tests were carried out using the statistics package R (R Core Team, 2017) to run paired Student’s *t*-tests on data from different wheat lines (results obtained using the same *Z. tritici* isolate in the same experimental set were treated as paired) using standard R commands for this function. The large numbers of *Z. tritici* isolates tested against the wheat genotypes of interest allowed for statistical assessments of the average broad resistance of each line. ANOVA tests were used when data from multiple wheat lines was to be compared, and to verify results produced from the *t*-tests – this was done using standard R and Excel Data Analysis commands.

## Results

### The assessment of multiple phenotypic traits for a large panel of *Z. tritici* isolate – wheat genotype interactions

Seventeen wheat genotypes carrying no known *Stb* genes, a single *Stb* gene, or a combination of *Stb* genes were screened against up to 100 current UK *Z. tritici* isolates. The symptoms of each genotype were compared to those of KWS Cashel, used as the susceptible control. The *P*-values derived using a standard student’s *t*-test to compare the average % pycnidia coverage of inoculated leaf area for each *Z. tritici* isolate-resistant wheat line to the equivalent averages from interactions with the KWS Cashel susceptible control are shown in **Table 2** – these data show which lines have significantly different symptom development levels overall compared to KWS Cashel (*P*<0.05). Mean average values the full set of genotype-isolate comparisons tested on each wheat line are given in **Table 3** for each of the four measured traits. The proportion of isolate-wheat line interactions for which disease symptoms were entirely absent for chlorosis/necrosis and for pycnidia development is shown in **Table 4**.

**Table 2:** A comparison of the % pycnidia coverage of inoculated leaf area for each *Z. tritici* isolate-resistant wheat genotype interaction and the equivalent values derived from the *Z. tritici* isolate’s interactions with the KWS Cashel susceptible control.

Wheat Genotype	*P*-value
**Taichung 29**	**1.5 × 10^-5^**
**Riband**	**3 × 10^-4^**
**Synthetic 6X**	**4.6 × 10^-12^**
**Synthetic M3**	**1.6 × 10^-10^**
**Kavkaz-K4500**	**4 × 10^-13^**
**Tadinia**	**7.3 × 10^-8^**
**Estanzuela Federal**	**1 × 10^-10^**
**Israel 493**	**7.2 × 10^-16^**
**TE9111**	**5.8 × 10^-18^**
**Bulgaria 88**	**2.1 × 10^-6^**
**Veranopolis**	**1.9 × 10^-6^**
**Synthetic M6**	**4.4 × 10^-5^**
**Tonic**	**1.3 × 10^-2^**
**Salamouni**	**3 × 10^-4^**
**Balance**	**2.3 × 10^-6^**
**Lorikeet**	**5.9 × 10^-7^**

A mean average from each interaction (calculated using the standard function in excel) were compared to that with KWS Cashel using a two-tailed Student’s *t*-test from the excel data analysis tool. The *P*-values resulting from this analysis are shown. All interactions show significant differences to the KWS Cashel susceptible control.

**Table 3:** The average symptoms on inoculated leaves of each wheat genotype.

Wheat Genotype	Level of resistance	*Stb* genes	No. *Z. tritici* isolates tested	Average No. days to appearance of symptoms	Average No. of days to appearance of pycnidia	Average final % of inoculated leaf area covered by chlorosis/necrosis	Average final % of inoculated leaf area covered by pycnidia
Taichung 29	Low	*None known*	68	13.0	16.4	97	21
Riband	Low	*Stb15*	90	14.2	17.7	79	23
KWS Cashel	Low	*None known*	85	14.7	17.6	84	36
Synthetic 6X	High	*Stb5*	70	15.4	25.3	50	1
Synthetic M3	High	*Stb16q, Stb17*	44	15.7	No pycnidia developed*	34	0
Kavkaz-K4500	High	*Stb6, Stb7, Stb10, Stb12*	65	17.7	No pycnidia developed*	22	0
Tadinia	Intermediate	*Stb4, Stb6*	71	17.1	19.6	55	9
Estanzuella Federal	Low/Intermediate	*Stb7*	62	14.1	19.1	85	10
Israel 493	High	*Stb3, Stb6*	74	13.6	22.7	59	1
TE9111	High	*Stb6, Stb7, Stb11*	84	17.8	20.7	31	1
Bulgaria 88	Intermediate/High	*Stb1, Stb6*	38	16.8	25.0	50	3
Veranopolis	Intermediate	*Stb2, Stb6*	37	15.9	23.2	47	6
Synthetic M6	Intermediate	*Stb8*	41	16.5	22.7	57	10
Tonic	Low/Intermediate	*Stb9*	29	15.1	21.1	78	15
Salamouni	Intermediate	*Stb6, Stb13, Stb14*	31	17.3	22.8	50	3
Balance	Intermediate	*Stb6, Stb18*	46	16.6	23.7	61	3
Lorikeet	High	*Stb6, Stb19*	31	18.9	No pycnidia developed*	22	0

* No visible pycnidia at time of assessment.

**Table 4:** The proportion of *Z. tritici* isolates that did not generate symptoms of each type on each wheat genotype in any interaction.

Wheat genotype	Overall level of resistance	*Stb* genes	No. *Z. tritici* isolates tested	% *Z. tritici* isolates that did not induce chlorosis/necrosis	% *Z. tritici* isolates that did not sporulate
Taichung 29	Low	none known	68	0	29
Riband	Low	*Stb15*	90	0	14
KWS Cashel	Low	none known	85	0	15
Synthetic 6X	High	*Stb5*	70	11	93
Synthetic M3	High	*Stb16q, Stb17*	44	32	100
Kavkaz-K4500	High	*Stb6, Stb7, Stb10, Stb12*	65	26	97
Tadinia	Intermediate	*Stb4, Stb6*	71	3	48
Estanzuella Federal	Low to intermediate	*Stb7*	62	0	34
Israel 493	High	*Stb3, Stb6*	74	5	92
TE9111	High	*Stb6, Stb7, Stb11*	84	10	92
Bulgaria 88	Intermediate to high	*Stb1, Stb6*	38	0	74
Veranopolis	Intermediate	*Stb2, Stb6*	37	3	68
Synthetic M6	Intermediate	*Stb8*	41	5	41
Tonic	Low to intermediate	*Stb9*	29	0	45
Salamouni	Intermediate	*Stb6, Stb13, Stb14*	31	3	68
Balance	Intermediate	*Stb6, Stb18*	46	0	80
Lorikeet	High	*Stb6, Stb19*	31	35	100

Inoculated wheat plants were assessed for four STB disease associated traits: the times (dpi) taken to the development of chlorosis/necrosis symptoms and fungal pycnidia, the final percentage of the inoculated leaf sections covered by chlorosis/necrosis and the final percentage of the inoculated leaf sections covered by pycnidia. Attempts were also made to quantify fungal sporulation in the inoculated leaves at 28 days post inoculation using spectrophotometry, but the obtained data was considered unreliable due to systemic over-estimation of spores by this method and was thus omitted for clarity.

#### Trait 1: Time to appearance of first symptoms

The time to the appearance of symptoms development for each seedling was measured as the number of days taken from inoculation to the first visible chlorosis or necrosis on the inoculated leaf area. There was significant biological variation in the rates of development of chlorosis and necrosis symptoms and percentage of leaf coverage by chlorosis/necrosis in some wheat line – *Z. tritici* isolate interactions (potentially caused by variation in factors such as sunlight levels, natural senescence or mechanical damage done during inoculation). This trait is therefore considered the least reliable indicator of fungal virulence of presented here. The wheat genotype that showed chlorosis/necrosis symptoms soonest on average was Taichung 29 at just 13 days post inoculation (dpi), although Israel 493 and Estanzuella Federal were close to this (13.6 and 14.1 dpi, respectively). The slowest average development of infection symptoms was in Lorikeet, with an average of 18.9 dpi.

#### Trait 2: Time to appearance of first pycnidia

The time to the appearance of pycnidia for each seedling was measured as the number of days taken from inoculation to the first visible pycnidia on the inoculated leaf area. The lowest average time to the appearance of pycnidia was 16.4 dpi in the wheat genotype Taichung 29. This value could not be obtained for Synthetic M3, Kavkaz-K4500 or Lorikeet due to the complete lack of pycnidia development in these genotypes. It should be noted that as this trait was not usually measurable in incompatible (resistance) interactions, the values provided for this apply only to interactions that enabled some level of pycnidia formation.

#### Trait 3: Inoculated leaf coverage by symptoms

**Figure 1:**
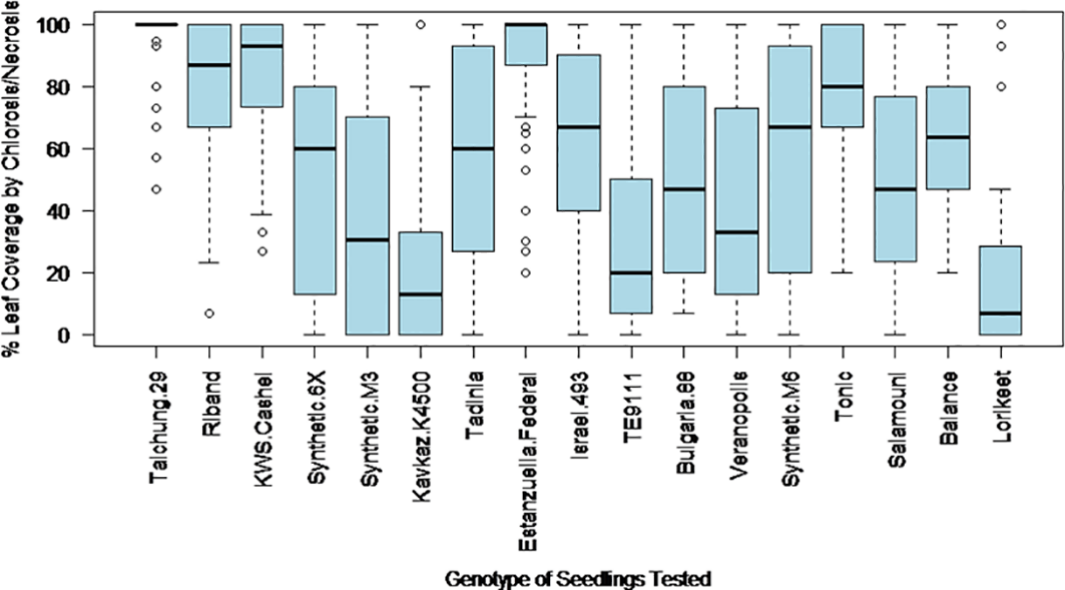
The variation in leaf coverage by chlorosis and necrosis induced by different *Z. tritici* isolates as observed at 28 days post inoculation on each of the 17 studied wheat genotypes.

The final percentage of the inoculated area of the leaf covered by chlorosis and necrosis at 28 days post inoculation was expected to provide an estimate of the relative levels of photosynthetic loss that could be expected from each wheat genotype when challenged with an isolate of *Z. tritici*. This trait showed high levels of variation both within and between wheat genotypes (**Figure 1**). Only highly resistant or highly susceptible genotypes showed more restricted ranges, with Estanzuella Federal leaves having consistently high symptoms coverage and leaves of Kavkaz-K4500 displaying consistently lower symptoms coverage. Due to these high ranges in the results obtained from most genotypes and the potential for occasional leaf damage due to the inoculation procedure (leading to the overestimation of symptoms), this phenotypic trait was considered less reliable than Trait 4.

#### Trait 4: Inoculated leaf coverage by pycnidia

**Figure 2:**
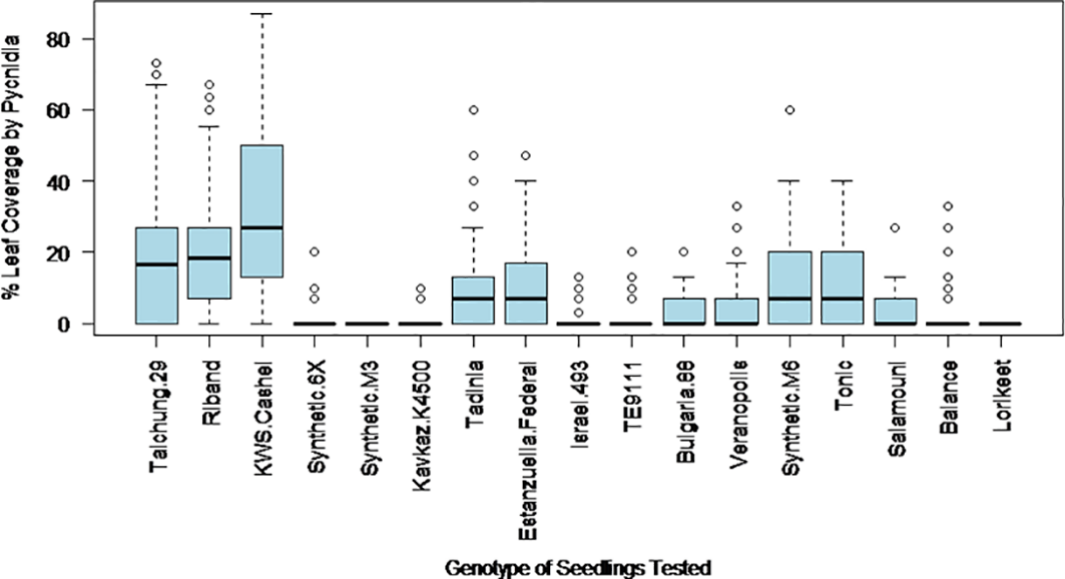
The variation in leaf coverage by pycnidia at 28 days post inoculation with different *Z. tritici* isolates on each of the 17 studied wheat genotypes.

The final percentage of the inoculated area of the leaf covered by pycnidia at 28 days post inoculation was expected to provide an estimate of the extent to which each isolate of the pathogen could effectively complete its asexual reproductive cycle on each wheat genotype, which is likely to be the strongest measured indicator of the capacity of each isolate to generate an epidemic in the field. The percentage of leaf area covered by pycnidia was more consistent for wheat genotype – *Z. tritici* isolate interactions than Trait 3, and thus became the primary factor used to differentiate between disease resistance and susceptibility. The variation in pycnidia coverage levels for each wheat genotype over the range of *Z. tritici* isolates tested is shown in **Figure 2**. The highest average level of pycnidia coverage was 36% in KWS Cashel, while the lowest were 0% for Synthetic M3, Kavkaz-K4500 and Lorikeet.

The percentage leaf coverage by pycnidia in all other tested genotypes was significantly lower compared to the susceptible control KWS Cashel in two-tailed paired Student’s *t*-tests (**Table 2**). This includes Taichung 29, which contains no known *Stb* genes. This may be due to possible differences in the plant leaf architecture resulting in fewer fungal penetration events, or potentially due to previously unidentified minor-effect resistance QTL(s). This indicates that all other wheat genotypes tested were significantly more resistant than KWS Cashel using this phenotypic trait, which is most directly connected to these isolates’ ability to cause an epidemic in the field.

### Comparative assessment of average levels of *Z. tritici* resistance in wheat genotypes based on four phenotypic traits

It should be emphasised that these results are calculated by averaging disease assessment scores from many individual *Z. tritici* isolates tested for each wheat genotype. Resistant genotypes, such as TE9111, Kavkaz-K4500 and Synthetic 6X were generally resistant to almost all isolates tested. However, genotypes, such as Tadinia had far more variable resistance, with some isolates inducing high infection scores across all assessment criteria while others produced no symptoms, generating intermediate average scores (**Table 3**). This suggests that these resistances are specific to fungal isolates carrying particular avirulence factors (a “gene-for-gene” relationship) which are each present in only some UK *Z. tritici* isolates. This also indicates that the underlying resistance mechanisms are highly effective when recognition occurs early in *Z. tritici* development, even against isolates with the potential to be highly virulent on other lines.

In most cases, wheat genotypes displayed similar symptom severity across all measurements. However, for some genotypes (e.g. Israel 493) the development rate and final percentage leaf coverage of chlorosis were high compared to the final percentage of pycnidia leaf coverage. Similarly, early chlorosis followed by high resistance to pycnidia development were seen in Synthetic 6X and Synthetic M3, although not all *Z. tritici* isolates stimulated visible chlorosis development in these lines (e.g. RResHT-8 and RResHT-10 induced 33-86% chlorosis in both Synthetic 6X and Synthetic M3, whereas RResHT-21 and RResHT-24 generated 0-7% chlorosis in both lines).

The results obtained in this study demonstrate great variability between the resistances of different wheat lines to UK *Z. tritici* isolates. As expected, wheat lines containing no known *Stb* genes are by far the least resistant group, with almost all tested isolates being highly virulent against KWS Cashel and Taichung 29. This indicates the very low levels of non-specific resistance for *Z. tritici* present in most wheat lines.

Overall, in addition to the wheat genotypes Taichung 29 and KWS Cashel (no known *Stb* genes), Riband [*Stb15*-common and widely broken in Europe (Arraiano et al., 2009)] was more susceptible than other lines. Estanzuela Federal (*Stb7*) also showed low resistance to most isolates tested (though higher than in fully susceptible lines for pycnidia coverage), indicating that UK *Z. tritici* populations are virulent towards *Stb7* and *Stb15*. Tonic also showed relatively low resistance although it was less susceptible than Taichung 29, KWS Cashel or Riband.

Israel 493 (*Stb3* and *Stb6*) and TE9111 (*Stb6, Stb7* and *Stb11*) showed relatively high levels of resistance, indicating that *Stb3* and *Stb11* could be of high potential interest to UK breeders. The synthetic and synthetic-derived lines Synthetic 6X, Synthetic M3 and Lorikeet also demonstrated high levels of resistance, likely due to their novel *Stb* resistance genes (*Stb5*, *Stb16q* and *Stb17*, and *Stb19* respectively). Kavkaz-K4500 (*Stb6*, *Stb7, Stb10* and *Stb12*) provides good levels of resistance, likely due to the presence of *Stb10* and *Stb12* (as *Stb6* is known to be widely broken and *Stb7* has been shown to be ineffective due to the susceptibility of Estanzuela Federal).

The lines Tadinia, Balance, Synthetic M6, Bulgaria 88, Veranopolis, and Salamouni had more intermediate average levels of resistance, indicating that the genes *Stb1, Stb2, Stb4, Stb8, Stb9, Stb13, Stb14* and *Stb18* all provided partial resistance, or provided resistance to some but not all *Z. tritici* isolates tested. These *Stb* genes could also be interesting to breeders as most would take relatively little effort to move into new wheat cultivars, and are likely to produce reasonable levels of resistance under field conditions (where inoculum levels will be lower than in these screens). However, the genetic variability of *Z. tritici* in the field suggests that individually these genes are unlikely to offer stable resistance, as at least one *Z. tritici* isolate will be virulent against each. It is likely that these genes would have to be stacked to provide durable resistance, slowing and complicating the breeding process.

It was notable that Riband, Estanzuela Federal and Tonic possessed the least resistance among *Stb* gene containing genotypes. Riband showed the highest levels of pycnidia amongst the lines possessing at least one *Stb* gene. This is likely to be because *Stb15* is known to have been widely present in European wheat lines historically (Arraiano et al., 2009), meaning that the local *Z. tritici* populations have adapted to its presence. Tonic had the second highest levels of pycnidiospore production and Estanzuela Federal having the second highest levels of pycnidia coverage. This suggests that the *Stb* genes found in these lines (*Stb7*, *Stb9* and *Stb15*) do not provide good resistance to most *Z. tritici* isolates present in the UK population and should be considered low priority breeding targets for UK wheat lines (although these genes may be more effective against *Z. tritici* populations in other parts of the world).

### Identification of preferential breeding targets for maximising the durability of STB resistance genes

The broadest complete resistances were found in Synthetic M3, Kavkaz-K4500, TE9111 and Lorikeet. These genotypes collectively contain *Stb6, Stb7, Stb10, Stb11, Stb12, Stb16q, Stb17*, and *Stb19*. However, the *Z. tritici* isolates used in this test were selected from a dataset of isolates known to be virulent against lines containing *Stb6*. Additionally, *Stb6* and *Stb7* were present in less resistant lines (e.g. Veranopolis and Estanzuela Federal), likely indicating that these *Stb* genes contributed minimally to the resistances of these cultivars.

In Kavkaz-K4500 and Synthetic M3, *Stb10* is paired with *Stb12* and *Stb16q* is paired with *Stb17*, respectively. As none of the genotypes tested contained these genes individually, it is difficult to determine from these results what proportion of the resistances each gene in these pairs was responsible for. It should be noted that previous experiments and field observations demonstrate that *Stb16q* provides extremely broad resistance to the UK *Z. tritici* population present in 2015-2017 (Tabib Ghaffary et al., 2012; Saintenac et al., 2021) whilst *Stb17* was demonstrated to act primarily in adult plants, older than the seedlings used in this study (Tabib Ghaffary et al., 2012), indicating that *Stb16q* is likely to be responsible for most of the resistance seen in Synthetic M3.

Further experimentation using nearly isogenic lines containing each of these genes individually will aid determining for certain which provide the broadest resistance – until such time as this work is completed, *Stb5*, *Stb11* and *Stb19* appear to be the highest priority breeding targets found in these bioassays.

### Identification of a class of STB resistance responses associated with strong early leaf chlorosis and reduced pycnidia production

**Figure 3:**
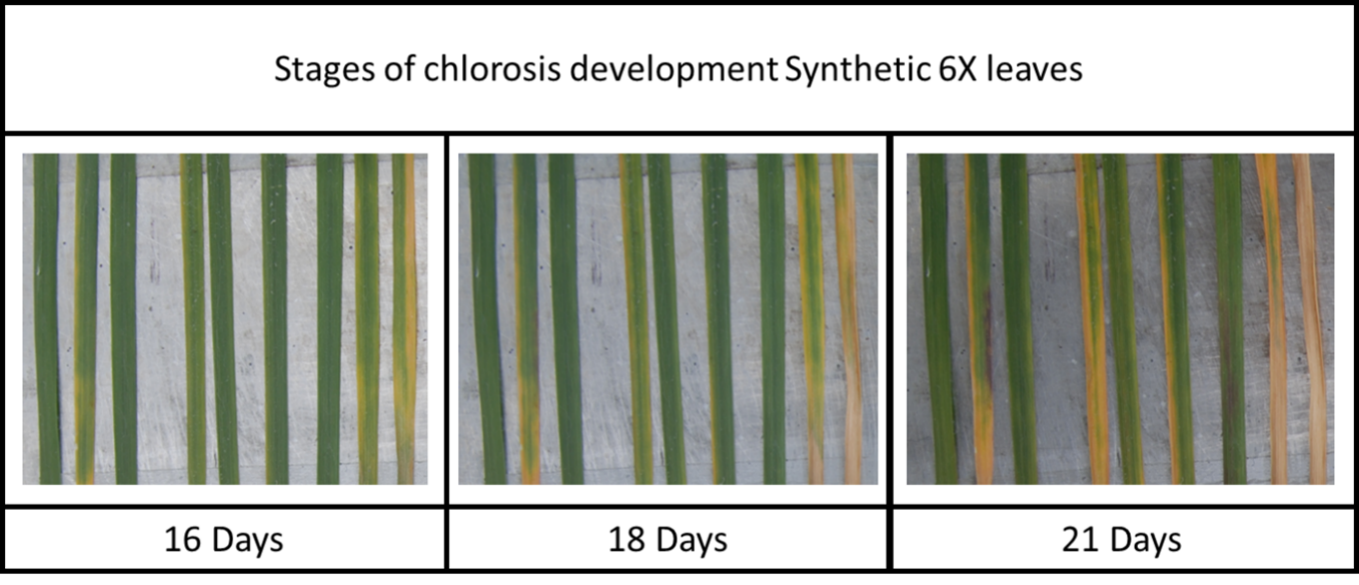
The early chlorosis symptoms and lack of fungal pycnidia observed on Synthetic 6X leaves at 28 days post inoculation with three different *Z. tritici* strains.

An examination of the level of resistance to different symptoms of *Z. tritici* infection in each wheat genotype also reveals a broader category of potentially interesting *Stb* genes that show high levels of resistance to pycnidia development but do not protect from the early development and high final coverages of chlorotic and necrotic symptoms on the leaves. For example, Israel 493 (containing *Stb3* and *Stb6*) shows the sixth highest average symptom coverage score of all tested genotypes (the fourth highest amongst genotypes possessing at least one *Stb* gene), yet has negligibly low average levels of pycnidia coverage, as shown in **Figure 3**. This could indicate the presence of resistance genes that act specifically to disrupt the pycnidia formation stage of fungal pathogen development or the presence of resistance pathways which cause chlorosis as a side effect less damaging then allowing the fungus to grow unimpeded, although it seems unlikely that chlorosis is directly tied to the resistance mechanism as chlorosis is usually linked with cell death and *Z. tritici* is primarily necrotrophic.

This unusual combination of symptoms could indicate the activation of resistance mechanisms involving a hypersensitive response, likely involving early reactive oxygen species-producing reactions in the chloroplasts (as indicated by the early and strong chlorosis response). This resistance mechanism seems likely to be effective at preventing the spread of a *Z. tritici* epidemic in the field by preventing pycnidia development, although there may also be some loss of photosynthetic potential from individual plants. This could suggest that *Stb3* and other resistance genes whose action is associated with high levels of chlorosis could provide more durable resistance if deployed in combination with other resistance genes, whose action is not associated with chlorosis, as the two different resistance mechanisms would be difficult for any *Z. tritici* isolate to adapt to. However, the utility of these resistances is likely to depend on the level of loss of photosynthetic potential in the field, which cannot easily be estimated from this work, as the high levels of inoculum used to ensure infection here are unrealistic to occur under normal field conditions. Additionally, it is not known which resistance response would be activated against isolates avirulent on wheat genotypes containing both resistance genes associated with chlorosis and those that do not associate with chlorosis. Further experimentation and fieldwork are needed to determine the utility of combining these two mechanistically different types of resistance responses.

## Discussion

*Zymoseptoria tritici* is one of the most important pathogens in the wheat-based agricultural systems of Europe, and chemical defences against it do not seem likely to be durable in the long term. It is therefore vital that breeders be able to effectively utilise *Stb* resistance genes to prevent major epidemics. This study provides data that will help to target UK breeding efforts to the most effective *Stb* resistance genes.

Data provided by field trials can be difficult to standardise due to genetic differences in *Z. tritici* populations locally (Berraies et al., 2013; Mekonnen et al., 2020) and globally, and due to the dramatic effect of weather conditions (particularly rainfall) on STB disease development, which can cause large fluctuations in readings between years at the same sites (Ouaja et al., 2020). Additional complexities are added to data analysis by wheat lines with resistance levels that change over the wheat life cycle (e.g. high seedling and low adult resistance) and by imperfect correlations between the levels of different infection symptoms (e.g. necrosis levels and pycnidia counts) (Ouaja et al., 2020). This information is particularly lacking for novel STB disease resistance sources, such as synthetic hexaploid wheats. Overall, the results presented here suggest that the lines Lorikeet (containing *Stb19*) and Synthetic M3 (containing *Stb16q* and *Stb17*) should be of the greatest interest to breeders, as these genotypes were resistant to pycnidia formation from every *Z. tritici* isolate they were challenged with in our bioassays, along with Kavkaz-K4500 (containing *Stb6, Stb7, Stb10* and *Stb12*), Synthetic 6X (containing *Stb5*) and TE9111 (containing *Stb6*, *Stb7* and *Stb11*), which had very high overall resistance. However, Synthetic M3 carries two *Stb* genes, *Stb16q* and *Stb17*. Of these, previous research suggests that *Stb17* is effective only in adult plants (Tabib Ghaffary et al., 2012), suggesting that the Synthetic M3 resistance is primarily due to the effect of *Stb16q*, which is known to provide broad resistance against *Z. tritici*. However, it should be noted that the resistance provided by *Stb16q* in the field is likely to be less complete than these results suggest, as the bioassays described here used UK *Z. tritici* isolates collected between 2015 and 2017. Since these dates, use of *Stb16q* in elite wheat lines has led to selection for *Z. tritici* isolates capable of virulence against lines containing this resistance gene, e.g. those found in Ireland and Iran (Dalvand et al., 2018; Kildea et al., 2020), which will likely lead to reductions in the field effectiveness of *Stb16q* over the coming years (as has previously been seen for *Stb6* and *Stb15*). This effect has not yet been noted for the resistance gene *Stb19*, which has not been used in the UK thus far. However, it seems likely that wider use of *Stb19* in elite lines would favour the development of *Z. tritici* isolates capable of breaking this resistance, leading to the loss of efficacy of this resistance gene. It is therefore important that when *Stb19* is used, it is supported by additional genes that provide broad resistance to the local *Z. tritici* population.

The results of this bioassay suggest Kavkaz-K4500 (*Stb6, Stb7, Stb10* and *Stb12*), Synthetic 6X (*Stb5*) and TE9111 (*Stb6, Stb7* and *Stb11*) as good potential sources for these protective *Stb* resistance genes. These genotypes show no pycnidia development from 98%, 96% and 95% of tested *Z. tritici* isolates respectively, with low pycnidia coverages (a maximum of 20% average) from the remaining isolates. All isolates tested against all three genotypes proved avirulent against at least one. As results from Estanzuella Federal and previous research suggest that *Stb6* and *Stb7* provide little or no resistance from UK *Z. tritici* populations (Czembor et al., 2011; Makhdoomi et al., 2015; Stephens et al., 2021), it seems likely that *Stb5, Stb11* and either *Stb10* or *Stb12* are responsible for these resistances. As *Stb10* and *Stb12* were not available for testing in isolation, it was not possible in this study to assess proportion of the total Kavkaz-K4500 resistance associated with each of these genes. Therefore currently *Stb5* and *Stb11* appear to be the optimal resistances to protect the durability of *Stb19* in future wide use. The long-term effectiveness of the Kavkaz-K4500 resistance despite the widespread use of this genotype in breeding suggests that such pyramids of mutually protective *Stb* genes are likely to be effective in slowing the development of virulence against them in *Z. tritici* populations.

The most useful *Stb* genes identified here are novel genes originating from synthetic hexaploid wheat lines and those that have historically been protected by the presence of multiple resistances in a single breeding line. This may cause issues during the breeding process, as synthetic-derived lines could carry undesirable genes (causing linkage drag when resistances are transferred to elite lines, possibly reducing yields) and effective resistances may be difficult to identify from wheat lines in which they coexist with several ineffective resistances. The high average resistance of novel lines aligns well with the results of (Arraiano and Brown, 2006), which found that of 238 wheat genotypes tested, the line with the highest non-specific resistance in their study was the Italian landrace Rieti. Although the resistances identified as broadly effective in this study were highly specific rather than non-specific, both results still indicate that the time given for *Z. tritici* to adapt to widely used resistances is a vital determining factor in their effectiveness. However, the (Arraiano and Brown, 2006) paper utilised isolates, which are now severely outdated and several generations removed from current wild *Z. tritici* populations, along with detached leaf assays, which may cause issues with measuring symptoms such as necrosis coverage (which (Arraiano and Brown, 2006) did not attempt to monitor). This study used more recent field isolates of *Z. tritici* collected from a more localised region around the UK and tested against a smaller set of wheat genotypes, producing a dataset more optimally targeted for identifying resistance genes of interest to breeders in this area. This study also selected wheat genotypes for testing based on the presence of known major resistance genes whereas (Arraiano and Brown, 2006) aimed to test a broader set of wheat genotypes for any resistance regardless of genetic origin, which together with the more modern *Z. tritici* isolates used in the present study makes it difficult to draw direct conclusions from differences in the average resistances observed.

Resistance to *Z. tritici* is a relatively new target in wheat breeding, meaning that much of the research relating to this pathogen and its interactions with crop plants is still in the early stages and major details of the infection and resistance processes (e.g. potential *Z. tritici* effector impacts on host chloroplast function or the mechanisms of most *Stb* gene-for-gene resistances) are largely unknown at a molecular level. Up so far, only *Stb6* and *Stb16q* have been cloned (along with the corresponding fungal effector AvrStb6 recognised by *Stb6*) (Zhong et al., 2017; Saintenac et al., 2018; Saintenac et al., 2021). Much of the research conducted thus far has utilised the model isolate held by most laboratories, IPO 323 – however, this isolate is not reflective of modern field isolates in important ways. For example, IPO 323 is naïve to all modern fungicides and avirulent on cultivars with disease resistance genes that have now been broken down by a large majority of isolates found in the field (e.g. *Stb6*). It is therefore important that novel *Stb* resistance genes be tested more broadly against collections rather than single *Z. tritici* isolates, to assess whether they act sufficiently broadly to be useful in a commercial growing context. The *Z. tritici* isolates utilised in this study were selected from UK fields between the years 2015 and 2017, and are virulent against *Stb6*. Although these isolates have not been sequenced, the range of different resistance responses they triggered in some wheat genotypes suggests a high level of genetic diversity. This is supported by the well-established genetic diversity of *Z. tritici* even in limited geographic regions (Berraies et al., 2013; Mekonnen et al., 2020; Orellana-Torrejon et al., 2022b) and indicates that the results identified here should be broadly applicable to UK *Z. tritici* populations.

Although broadly resistant wheat genotypes shared resistance to some specific *Z. tritici* isolates with each other, no statistically significant associations were found between the specific isolates that were included in this resistance and those which remain virulent against each host genotype (data not shown). This suggests that most of the *Stb* resistance genes tested here operate through the recognition of different avirulence factors. No *Z. tritici* isolate tested here was shown to be virulent against all host genotypes assessed in this study. Therefore, it should be possible to develop highly resistant breeding lines by stacking many *Stb* genes. Such gene pyramids would likely improve the durability of all *Stb* genes included (provided that these *Stb* genes were only used in such gene pyramids), as it is much less likely that any given isolate would gain all of the required mutations for virulence at once and thus overcome the resistance. This could be extremely useful in the long term – for example, Kavkaz-K4500 has been considered an STB resistant breeding line for many years and still appeared effective in our experiments, suggesting that combinations of resistance genes that utilise different mechanisms may not only help to increase the durability of each individual gene, but could also be broadly effective due to the collective action of these genes. The use of modern genetic markers and breeding techniques will be necessary to overcome potential obstacles to breeding such as linkage drag and epistasis effects – for example, markers could help track specific resistance genes present in breeding materials derived from genotypes containing multiple *Stb* genes, and the production of nearly isogenic lines assisted through genotyping using such markers could limit the effect of linkage drag on new breeding lines. However, it is likely that significant breeding work would still be required to introgress the majority of the *Stb* genes examined here into the regionally adapted elite breeding lines, as the corresponding disease resistance sources used in this study were originally bred for different environments and growth habits (e.g. Bulgaria 88 is a Bulgarian winter type wheat, whereas Israel 493 is an Israeli spring type wheat) and most are not recent but were developed years or decades ago.

In summary, this study revealed that sufficiently diverse *Stb* genes exist to give broad and durable protection from UK *Z. tritici* isolates to new wheat lines. However, generating this protection in a sustainable form will require extensive breeding efforts. We identified suitable *Stb* genes to prioritise for pyramiding. However, further work will be necessary to identify modern high-throughput markers such as Kompetitive Allele Specific PCR (KASP) markers (Semagn et al., 2014) for each *Stb* gene of interest to ensure that multiple broadly effective genes can be stacked in a single line (as otherwise epistatic effects may make their presence difficult to confirm), and to produce lines containing each *Stb* gene from highly resistant lines individually for further detailed characterization. There therefore remains much work to be done collaboratively between UK wheat breeders and the scientific community to ensure the desired level of resistance in future wheat.

## Data availability statement

The original contributions presented in the study are included in the article/**Supplementary Material**. Further inquiries can be directed to the corresponding authors.

## Author contributions

All authors contributed to the writing of this paper, with HT acting as lead writer. KK and RB conceived this project and contributed materials to this project. HT acted as primary experimenter and performed measurement, compilation and statistical analysis of results. All authors contributed to the article and approved the submitted version.
