# Supplementary material for: A large bioassay identifies Stb resistance genes that provide broad resistance against Septoria tritici blotch disease in the UK
Source: Front Plant Sci. 2023 Jan 9;13:1070986. doi: 10.3389/fpls.2022.1070986 (PMC9868401; doi:10.3389/fpls.2022.1070986)
Supplement: Supplementary file 1 [file DataSheet_1.docx]

**Supplementary Figures**

**Supplementary Figure 1:** Experimental set up for high humidity incubation of seedlings during the first 72 hours post inoculation with *Zymoseptoria tritici*


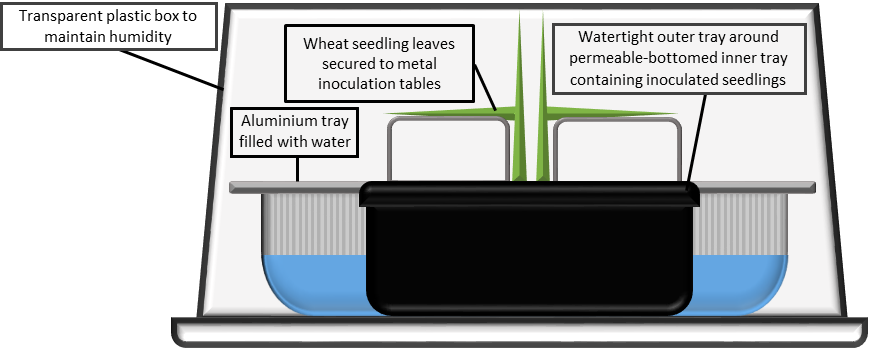


**Supplementary Figure 2:** Average percentage of inoculated leaf area covered by necrosis and chlorosis on 17 different wheat genotypes at 28 days post inoculation for *Z. tritici* isolates sourced from different wheat varieties (indicated on the X-axis) or the reference isolate IPO 323. A single-factor ANOVA test shows that the variations between the average symptom coverages caused by *Z. tritici* isolates sourced from the different wheat genotype are significant (*p*=0.001).


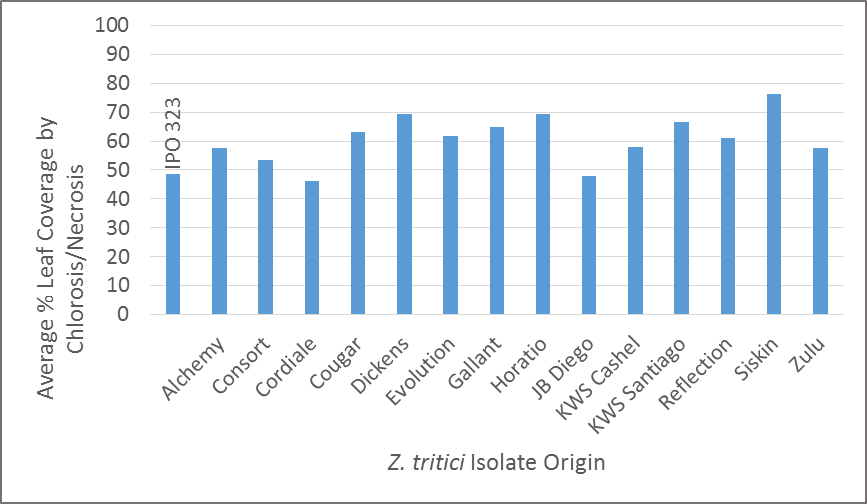


**Supplementary Figure 3:** Average percentage of inoculated leaf area covered by pycnidia on 17 different wheat genotypes at 28 days post inoculation for *Z. tritici* isolates sourced from different wheat varieties (indicated on the X-axis) or the reference isolate IPO 323. A single-factor ANOVA test shows that the variations between the average symptom coverages caused by *Z. tritici* isolates sourced from the different wheat genotype are not significant (*p*=0.114).


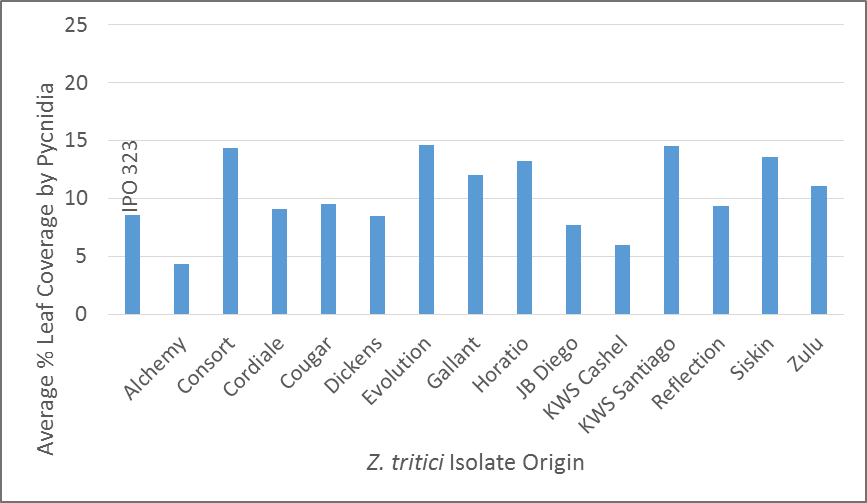


**Supplementary Table 1:** Full data showing which wheat genotypes could be classified as resistant or susceptible based on the % leaf area covered by chlorosis/necrosis at 28 days post inoculation with different *Zymoseptoria tritici* isolates. Interactions classified as resistant failed to produce any visible chlorosis or necrosis symptoms across the tested leaves. Missing data is represented with a dash.

| *Z. tritici* isolate | Taichung 29 | Riband | KWS Cashel | Synthetic 6X | Synthetic M3 | Kavkaz K4500 | Tadinia | Estanzuella Federal | Israel 493 | TE9111 | Bulgaria 88 | Veranopolis | Synthetic M6 | Tonic | Salamouni | Balance | Lorikeet |
| --- | --- | --- | --- | --- | --- | --- | --- | --- | --- | --- | --- | --- | --- | --- | --- | --- | --- |
| IPO 323 | Susceptible | Susceptible | Susceptible | Susceptible | Susceptible | Susceptible | Susceptible | Susceptible | Susceptible | Susceptible | Susceptible | Susceptible | Susceptible | Susceptible | Susceptible | Susceptible | Susceptible |
| RResHT-1 | Susceptible | Susceptible | Susceptible | Susceptible | Resistant | Resistant | Susceptible | Susceptible | Susceptible | Susceptible | - | - | - | - | - | - | - |
| RResHT-2 | Susceptible | Susceptible | Susceptible | Susceptible | Susceptible | Resistant | Susceptible | Susceptible | Susceptible | Susceptible | Susceptible | Susceptible | Susceptible | Susceptible | Susceptible | Susceptible | - |
| RResHT-3 | Susceptible | Susceptible | Susceptible | Susceptible | - | Resistant | Susceptible | Susceptible | Resistant | Susceptible | - | - | - | - | - | Susceptible | - |
| RResHT-4 | Susceptible | Susceptible | Susceptible | Resistant | Susceptible | Susceptible | Susceptible | Susceptible | Susceptible | Susceptible | - | - | - | - | - | - | - |
| RResHT-5 | Susceptible | Susceptible | Susceptible | Susceptible | - | Susceptible | Susceptible | Susceptible | Susceptible | Resistant | - | - | - | - | - | Susceptible | - |
| RResHT-6 | Susceptible | Susceptible | Susceptible | Susceptible | Resistant | Susceptible | Susceptible | Susceptible | Susceptible | Susceptible | - | - | - | - | - | - | - |
| RResHT-7 | Susceptible | Susceptible | Susceptible | Susceptible | - | Resistant | Susceptible | Susceptible | Susceptible | Resistant | - | - | - | - | - | Susceptible | - |
| RResHT-8 | Susceptible | Susceptible | Susceptible | Susceptible | Susceptible | Susceptible | Susceptible | Susceptible | Susceptible | Susceptible | Susceptible | Susceptible | Susceptible | Susceptible | Susceptible | Susceptible | - |
| RResHT-9 | Susceptible | Susceptible | Susceptible | Susceptible | Susceptible | Susceptible | Susceptible | - | Susceptible | Susceptible | - | - | - | - | - | - | - |
| RResHT-10 | Susceptible | Susceptible | Susceptible | Susceptible | Susceptible | Susceptible | Susceptible | Susceptible | Susceptible | Susceptible | Susceptible | Susceptible | Susceptible | Susceptible | Susceptible | Susceptible | - |
| RResHT-11 | Susceptible | Susceptible | Susceptible | - | - | - | - | Susceptible | Susceptible | Susceptible | - | - | - | - | - | - | - |
| RResHT-12 | Susceptible | Susceptible | Susceptible | Susceptible | - | Susceptible | Susceptible | - | Susceptible | Susceptible | - | - | - | - | - | Susceptible | - |
| RResHT-13 | Susceptible | Susceptible | Susceptible | Susceptible | - | Resistant | Susceptible | Susceptible | Susceptible | Susceptible | - | - | - | - | - | - | - |
| RResHT-14 | Susceptible | Susceptible | Susceptible | Susceptible | Resistant | - | Susceptible | Susceptible | Susceptible | Susceptible | Susceptible | Susceptible | Susceptible | Susceptible | Susceptible | - | - |
| RResHT-15 | Susceptible | Susceptible | Susceptible | Susceptible | - | Susceptible | Susceptible | Susceptible | Susceptible | Susceptible | - | - | - | - | - | Susceptible | - |
| RResHT-16 | Susceptible | Susceptible | Susceptible | Susceptible | - | - | Susceptible | Susceptible | Susceptible | Susceptible | Susceptible | Susceptible | Susceptible | - | - | - | - |
| RResHT-17 | Susceptible | Susceptible | Susceptible | - | - | - | - | Susceptible | Susceptible | Susceptible | - | - | - | - | - | - | - |
| RResHT-18 | Susceptible | Susceptible | Susceptible | Susceptible | - | Susceptible | Susceptible | Susceptible | Susceptible | Susceptible | - | - | - | - | - | - | - |
| RResHT-19 | Susceptible | Susceptible | Susceptible | Susceptible | - | Resistant | Susceptible | Susceptible | Susceptible | Susceptible | - | - | - | - | - | - | - |
| RResHT-20 | Susceptible | - | - | Susceptible | Susceptible | Susceptible | Susceptible | Susceptible | - | Susceptible | Susceptible | Susceptible | Susceptible | Susceptible | - | Susceptible | - |
| RResHT-21 | Susceptible | Susceptible | Susceptible | Resistant | Resistant | Susceptible | Susceptible | Susceptible | Resistant | Susceptible | - | - | - | - | - | - | - |
| RResHT-22 | Susceptible | Susceptible | Susceptible | Susceptible | - | Susceptible | Susceptible | Susceptible | Resistant | Susceptible | - | - | - | - | - | Susceptible | - |
| RResHT-23 | Susceptible | Susceptible | Susceptible | Susceptible | - | Susceptible | Susceptible | Susceptible | Susceptible | Susceptible | - | - | - | - | - | - | - |
| RResHT-24 | Susceptible | Susceptible | Susceptible | Susceptible | Susceptible | Susceptible | Susceptible | Susceptible | Susceptible | Resistant | Susceptible | Susceptible | Susceptible | Susceptible | Susceptible | Susceptible | Susceptible |
| RResHT-25 | Susceptible | Susceptible | Susceptible | Susceptible | Resistant | Resistant | Susceptible | Susceptible | Susceptible | Resistant | - | - | - | - | - | - | - |
| RResHT-26 | Susceptible | Susceptible | Susceptible | Susceptible | - | Susceptible | Susceptible | Susceptible | Susceptible | Resistant | - | - | - | - | - | Susceptible | - |
| RResHT-27 | Susceptible | Susceptible | Susceptible | Susceptible | - | Resistant | Susceptible | Susceptible | Susceptible | Susceptible | - | - | - | - | - | - | - |
| RResHT-28 | Susceptible | Susceptible | Susceptible | Susceptible | Susceptible | Susceptible | Susceptible | Susceptible | Susceptible | Susceptible | - | - | - | - | - | - | - |
| RResHT-29 | Susceptible | Susceptible | Susceptible | Resistant | Susceptible | Susceptible | Susceptible | Susceptible | Susceptible | Susceptible | Susceptible | Susceptible | Susceptible | Susceptible | Susceptible | Susceptible | Susceptible |
| RResHT-30 | Susceptible | Susceptible | Susceptible | Susceptible | Susceptible | Susceptible | Susceptible | - | - | Susceptible | Susceptible | Susceptible | Susceptible | - | Susceptible | Susceptible | - |
| RResHT-31 | Susceptible | Susceptible | Susceptible | Susceptible | - | - | Susceptible | - | - | Susceptible | Susceptible | Susceptible | Susceptible | - | - | - | - |
| RResHT-32 | - | Susceptible | Susceptible | - | - | - | - | - | - | Susceptible | - | - | - | - | - | - | - |
| RResHT-33 | - | Susceptible | Susceptible | Susceptible | - | - | Susceptible | - | - | Susceptible | - | - | - | - | - | - | - |
| RResHT-34 | Susceptible | Susceptible | Susceptible | Susceptible | Susceptible | Resistant | Resistant | Susceptible | Resistant | Susceptible | Susceptible | Susceptible | Susceptible | Susceptible | Susceptible | Susceptible | Resistant |
| RResHT-35 | - | Susceptible | Susceptible | Resistant | Susceptible | Susceptible | Susceptible | - | - | Resistant | - | - | - | - | - | - | - |
| RResHT-36 | - | Susceptible | Susceptible | Susceptible | - | Resistant | Susceptible | - | - | Resistant | - | - | - | - | - | Susceptible | - |
| RResHT-37 | Susceptible | Susceptible | Susceptible | Susceptible | Susceptible | Resistant | Susceptible | Susceptible | Susceptible | Susceptible | Susceptible | Susceptible | Susceptible | Susceptible | Susceptible | Susceptible | Susceptible |
| RResHT-38 | Susceptible | Susceptible | Susceptible | Susceptible | Susceptible | Resistant | Resistant | Susceptible | Susceptible | Susceptible | Susceptible | Susceptible | Susceptible | Susceptible | Susceptible | Susceptible | Susceptible |
| RResHT-39 | Susceptible | Susceptible | Susceptible | Susceptible | Susceptible | Resistant | Susceptible | Susceptible | Susceptible | Susceptible | Susceptible | Susceptible | Resistant | Susceptible | Resistant | Susceptible | Resistant |
| RResHT-41 | - | Susceptible | Susceptible | Susceptible | - | Resistant | Susceptible | - | - | Susceptible | - | - | - | - | - | Susceptible | - |
| RResHT-42 | - | Susceptible | Susceptible | Susceptible | - | Susceptible | Susceptible | - | - | - | - | - | - | - | - | Susceptible | - |
| RResHT-44 | Susceptible | Susceptible | Susceptible | - | Susceptible | Susceptible | Susceptible | Susceptible | - | Susceptible | Susceptible | Susceptible | Susceptible | Susceptible | Susceptible | Susceptible | Resistant |
| RResHT-45 | Susceptible | Susceptible | Susceptible | Susceptible | Resistant | Susceptible | Susceptible | Susceptible | Susceptible | Susceptible | Susceptible | Susceptible | Susceptible | Susceptible | Susceptible | Susceptible | Resistant |
| RResHT-46 | - | Susceptible | - | - | - | - | - | - | Susceptible | - | - | - | - | - | - | - | - |
| RResHT-47 | - | Susceptible | Susceptible | Susceptible | - | Susceptible | Susceptible | - | Susceptible | - | - | - | - | - | - | Susceptible | - |
| RResHT-48 | - | Susceptible | Susceptible | Resistant | - | Resistant | - | - | Susceptible | Susceptible | - | - | - | - | - | - | - |
| RResHT-49 | - | Susceptible | - | - | - | - | - | - | Susceptible | Susceptible | - | - | - | - | - | - | - |
| RResHT-50 | - | Susceptible | Susceptible | Susceptible | - | Susceptible | Susceptible | - | - | - | - | - | - | - | - | Susceptible | - |
| RResHT-51 | Susceptible | Susceptible | Susceptible | Resistant | Resistant | Susceptible | Susceptible | Susceptible | Susceptible | Susceptible | Susceptible | Susceptible | Susceptible | Susceptible | Susceptible | Susceptible | Resistant |
| RResHT-52 | Susceptible | Susceptible | Susceptible | Susceptible | Resistant | Susceptible | Susceptible | Susceptible | Susceptible | Susceptible | Susceptible | Resistant | Susceptible | Susceptible | Susceptible | Susceptible | Resistant |
| RResHT-53 | Susceptible | Susceptible | Susceptible | Susceptible | Resistant | Susceptible | Susceptible | - | Susceptible | Susceptible | Susceptible | Susceptible | Susceptible | Susceptible | Susceptible | Susceptible | Resistant |
| RResHT-54 | - | Susceptible | Susceptible | Resistant | - | - | - | - | Susceptible | Susceptible | - | - | - | - | - | - | - |
| RResHT-55 | - | Susceptible | Susceptible | Susceptible | Susceptible | Susceptible | Susceptible | - | - | - | - | - | - | - | - | - | - |
| RResHT-56 | - | Susceptible | - | - | - | - | - | - | - | - | - | - | - | - | - | - | - |
| RResHT-57 | - | Susceptible | - | - | - | - | - | - | Susceptible | - | - | - | - | - | - | - | - |
| RResHT-58 | - | Susceptible | - | - | - | - | - | - | Susceptible | Susceptible | - | - | - | - | - | - | - |
| RResHT-60 | Susceptible | Susceptible | Susceptible | Susceptible | Susceptible | Susceptible | Susceptible | Susceptible | Susceptible | Susceptible | Susceptible | Susceptible | Susceptible | Susceptible | Susceptible | Susceptible | Susceptible |
| RResHT-62 | Susceptible | Susceptible | Susceptible | Susceptible | Susceptible | Susceptible | Susceptible | Susceptible | Susceptible | Susceptible | Susceptible | Susceptible | Susceptible | Susceptible | Susceptible | Susceptible | Susceptible |
| RResHT-63 | Susceptible | Susceptible | Susceptible | Susceptible | Susceptible | Susceptible | Susceptible | Susceptible | Susceptible | Susceptible | Susceptible | Susceptible | Susceptible | Susceptible | Susceptible | Susceptible | Susceptible |
| RResHT-64 | Susceptible | - | - | Susceptible | Susceptible | - | Susceptible | - | - | Resistant | Susceptible | Susceptible | Susceptible | - | Susceptible | Susceptible | - |
| RResHT-65 | - | - | - | Susceptible | - | - | Susceptible | - | - | Susceptible | Susceptible | Susceptible | Resistant | - | - | - | - |
| RResHT-66 | Susceptible | Susceptible | Susceptible | Susceptible | Susceptible | Susceptible | Susceptible | Susceptible | Susceptible | Susceptible | Susceptible | Susceptible | Susceptible | - | Susceptible | Susceptible | Susceptible |
| RResHT-67 | Susceptible | Susceptible | Susceptible | Susceptible | - | Resistant | Susceptible | Susceptible | Susceptible | Susceptible | Susceptible | Susceptible | Susceptible | - | Susceptible | Susceptible | Susceptible |
| RResHT-68 | - | Susceptible | Susceptible | - | - | - | - | - | - | - | - | - | Susceptible | - | - | - | Susceptible |
| RResHT-69 | Susceptible | Susceptible | Susceptible | Susceptible | Susceptible | Susceptible | Susceptible | Susceptible | Susceptible | Susceptible | Susceptible | Susceptible | Susceptible | Susceptible | Susceptible | Susceptible | Susceptible |
| RResHT-70 | - | Susceptible | Susceptible | - | Susceptible | Susceptible | Susceptible | - | Susceptible | Susceptible | Susceptible | Susceptible | Susceptible | - | Susceptible | Susceptible | Susceptible |
| RResHT-71 | - | Susceptible | Susceptible | Susceptible | Susceptible | Susceptible | Susceptible | - | Susceptible | Susceptible | Susceptible | - | Susceptible | Susceptible | Susceptible | - | Susceptible |
| RResHT-72 | Susceptible | Susceptible | Susceptible | Susceptible | - | Susceptible | Susceptible | Susceptible | Susceptible | Susceptible | - | - | - | - | - | Susceptible | - |
| RResHT-73 | Susceptible | Susceptible | Susceptible | Susceptible | Resistant | Susceptible | Susceptible | Susceptible | Susceptible | Susceptible | Susceptible | Susceptible | Susceptible | Susceptible | Susceptible | Susceptible | Susceptible |
| RResHT-74 | Susceptible | Susceptible | Susceptible | Susceptible | - | Susceptible | Susceptible | Susceptible | Susceptible | Susceptible | - | - | - | - | - | Susceptible | - |
| RResHT-75 | Susceptible | Susceptible | Susceptible | - | - | - | - | Susceptible | Susceptible | Susceptible | - | - | - | - | - | - | - |
| RResHT-76 | Susceptible | Susceptible | Susceptible | - | Susceptible | Susceptible | - | Susceptible | Susceptible | Susceptible | - | - | Susceptible | - | - | - | Resistant |
| RResHT-78 | Susceptible | Susceptible | Susceptible | - | - | - | - | Susceptible | Susceptible | Susceptible | - | - | - | - | - | - | - |
| RResHT-79 | Susceptible | Susceptible | Susceptible | - | - | - | - | Susceptible | Susceptible | Susceptible | - | - | - | - | - | - | - |
| RResHT-80 | Susceptible | Susceptible | Susceptible | - | - | - | - | Susceptible | Susceptible | Susceptible | - | - | - | - | - | - | - |
| RResHT-82 | Susceptible | Susceptible | Susceptible | - | - | - | - | Susceptible | Susceptible | Susceptible | - | - | - | - | - | - | - |
| RResHT-83 | Susceptible | Susceptible | Susceptible | - | - | - | - | Susceptible | Susceptible | Susceptible | - | - | - | - | - | - | - |
| RResHT-84 | Susceptible | Susceptible | Susceptible | Susceptible | Susceptible | Susceptible | Susceptible | Susceptible | Susceptible | Susceptible | - | - | - | - | - | - | - |
| RResHT-85 | Susceptible | Susceptible | Susceptible | Resistant | - | Susceptible | Susceptible | Susceptible | Susceptible | Susceptible | - | - | - | - | - | - | - |
| RResHT-86 | Susceptible | Susceptible | Susceptible | - | - | - | - | Susceptible | Susceptible | Susceptible | - | - | - | - | - | - | - |
| RResHT-87 | Susceptible | Susceptible | Susceptible | - | - | - | - | Susceptible | Susceptible | Susceptible | - | - | - | - | - | - | - |
| RResHT-91 | - | Susceptible | Susceptible | Susceptible | - | - | Susceptible | - | Susceptible | Susceptible | - | - | - | - | - | - | - |
| RResHT-92 | - | Susceptible | Susceptible | Susceptible | - | - | Susceptible | - | - | Susceptible | - | - | - | - | - | - | - |
| RResHT-93 | - | Susceptible | Susceptible | - | - | - | - | - | - | Susceptible | - | - | - | - | - | - | - |
| RResHT-94 | - | Susceptible | - | - | - | - | - | - | - | Susceptible | - | - | - | - | - | - | - |
| RResHT-95 | Susceptible | Susceptible | Susceptible | Susceptible | Resistant | Susceptible | Susceptible | Susceptible | Susceptible | Susceptible | Susceptible | Susceptible | Susceptible | Susceptible | - | Susceptible | Susceptible |
| RResHT-96 | Susceptible | Susceptible | Susceptible | Susceptible | Resistant | Susceptible | Susceptible | Susceptible | Susceptible | Susceptible | Susceptible | Susceptible | Susceptible | Susceptible | - | Susceptible | Resistant |
| RResHT-97 | Susceptible | Susceptible | Susceptible | Susceptible | Resistant | Susceptible | Susceptible | Susceptible | Susceptible | Susceptible | Susceptible | Susceptible | Susceptible | - | Susceptible | Susceptible | Susceptible |
| RResHT-98 | Susceptible | Susceptible | Susceptible | Susceptible | Susceptible | Susceptible | Susceptible | Susceptible | Susceptible | Susceptible | Susceptible | Susceptible | Susceptible | Susceptible | Susceptible | Susceptible | Susceptible |
| RResHT-99 | Susceptible | Susceptible | Susceptible | Susceptible | Resistant | Resistant | Susceptible | Susceptible | Susceptible | Susceptible | Susceptible | Susceptible | Susceptible | Susceptible | Susceptible | Susceptible | Resistant |
| RResHT-100 | Susceptible | Susceptible | Susceptible | Susceptible | - | Susceptible | Susceptible | Susceptible | Susceptible | Susceptible | Susceptible | Susceptible | Susceptible | Susceptible | - | Susceptible | Susceptible |
| RResHT-101 | - | Susceptible | Susceptible | - | - | - | Susceptible | - | Susceptible | - | Susceptible | Susceptible | Susceptible | Susceptible | Susceptible | - | Susceptible |
| RResHT-102 | - | - | Susceptible | - | Susceptible | Susceptible | - | - | - | - | - | - | Susceptible | - | - | - | Resistant |

**Supplementary Table 2:** Full data showing which wheat genotypes could be classified as resistant or susceptible based on the % leaf area covered by pycnidia at 28 days post inoculation with different *Zymoseptoria tritici* isolates. Interactions classified as resistant failed to produce any visible pycnidia across the tested leaves. Missing data is represented with a dash.

| *Z. tritici* isolate | Taichung 29 | Riband | KWS Cashel | Synthetic 6X | Synthetic M3 | Kavkaz K4500 | Tadinia | Estanzuella Federal | Israel 493 | TE9111 | Bulgaria 88 | Veranopolis | Synthetic M6 | Tonic | Salamouni | Balance | Lorikeet |
| --- | --- | --- | --- | --- | --- | --- | --- | --- | --- | --- | --- | --- | --- | --- | --- | --- | --- |
| IPO 323 | Susceptible | Susceptible | Susceptible | Resistant | Resistant | Resistant | Susceptible | Susceptible | Susceptible | Resistant | Resistant | Susceptible | Resistant | Susceptible | Susceptible | Resistant | Resistant |
| RResHT-1 | Susceptible | Susceptible | Susceptible | Resistant | Resistant | Resistant | Resistant | Resistant | Resistant | Resistant | - | - | - | - | - | - | - |
| RResHT-2 | Susceptible | Susceptible | Susceptible | Resistant | Resistant | Resistant | Susceptible | Susceptible | Resistant | Resistant | Resistant | Resistant | Susceptible | Susceptible | Susceptible | Resistant | - |
| RResHT-3 | Susceptible | Susceptible | Susceptible | Susceptible | - | Resistant | Resistant | Resistant | Resistant | Resistant | - | - | - | - | - | Resistant | - |
| RResHT-4 | Susceptible | Susceptible | Susceptible | Resistant | Resistant | Resistant | Resistant | Susceptible | Resistant | Resistant | - | - | - | - | - | - | - |
| RResHT-5 | Susceptible | Susceptible | Susceptible | Resistant | - | Resistant | Resistant | Susceptible | Resistant | Resistant | - | - | - | - | - | Resistant | - |
| RResHT-6 | Susceptible | Susceptible | Susceptible | Resistant | Resistant | Resistant | Resistant | Susceptible | Resistant | Resistant | - | - | - | - | - | - | - |
| RResHT-7 | Susceptible | Susceptible | Susceptible | Resistant | - | Resistant | Resistant | Susceptible | Resistant | Resistant | - | - | - | - | - | Resistant | - |
| RResHT-8 | Susceptible | Susceptible | Susceptible | Resistant | Resistant | Resistant | Susceptible | Susceptible | Resistant | Resistant | Resistant | Resistant | Susceptible | Resistant | Susceptible | Resistant | - |
| RResHT-9 | Susceptible | Susceptible | Susceptible | Resistant | Resistant | Resistant | Resistant | - | Resistant | Resistant | - | - | - | - | - | - | - |
| RResHT-10 | Susceptible | Susceptible | Susceptible | Resistant | Resistant | Resistant | Susceptible | Susceptible | Resistant | Resistant | Susceptible | Resistant | Susceptible | Susceptible | Resistant | Resistant | - |
| RResHT-11 | Susceptible | Susceptible | Susceptible | - | - | - | - | Susceptible | Resistant | Resistant | - | - | - | - | - | - | - |
| RResHT-12 | Susceptible | Susceptible | Susceptible | Resistant | - | Resistant | Resistant | - | Resistant | Resistant | - | - | - | - | - | Resistant | - |
| RResHT-13 | Susceptible | Susceptible | Susceptible | Resistant | - | Resistant | Resistant | Resistant | Resistant | Resistant | - | - | - | - | - | - | - |
| RResHT-14 | Susceptible | Susceptible | Susceptible | Resistant | Resistant | - | Resistant | Susceptible | Resistant | Resistant | Resistant | Resistant | Resistant | Susceptible | Resistant | - | - |
| RResHT-15 | Susceptible | Susceptible | Susceptible | Resistant | - | Resistant | Resistant | Susceptible | Resistant | Resistant | - | - | - | - | - | Resistant | - |
| RResHT-16 | Susceptible | Susceptible | Susceptible | Resistant | - | - | Susceptible | Susceptible | Resistant | Resistant | Susceptible | Resistant | Susceptible | - | - | - | - |
| RResHT-17 | Susceptible | Susceptible | Resistant | - | - | - | - | Susceptible | Resistant | Resistant | - | - | - | - | - | - | - |
| RResHT-18 | Susceptible | Susceptible | Susceptible | Resistant | - | Resistant | Susceptible | Susceptible | Resistant | Susceptible | - | - | - | - | - | - | - |
| RResHT-19 | Susceptible | Resistant | Susceptible | Resistant | - | Resistant | Susceptible | Resistant | Resistant | Resistant | - | - | - | - | - | - | - |
| RResHT-20 | Susceptible | - | - | Resistant | Resistant | Resistant | Resistant | Susceptible | - | Resistant | Resistant | Resistant | Susceptible | Susceptible | - | Susceptible | - |
| RResHT-21 | Susceptible | Susceptible | Susceptible | Resistant | Resistant | Resistant | Resistant | Susceptible | Resistant | Resistant | - | - | - | - | - | - | - |
| RResHT-22 | Susceptible | Susceptible | Susceptible | Resistant | - | Resistant | Susceptible | Susceptible | Resistant | Susceptible | - | - | - | - | - | Resistant | - |
| RResHT-23 | Susceptible | Susceptible | Susceptible | Resistant | - | Resistant | Susceptible | Susceptible | Resistant | Resistant | - | - | - | - | - | - | - |
| RResHT-24 | Susceptible | Susceptible | Susceptible | Resistant | Resistant | Resistant | Susceptible | Susceptible | Resistant | Resistant | Susceptible | Susceptible | Susceptible | Resistant | Resistant | Resistant | Resistant |
| RResHT-25 | Susceptible | Susceptible | Susceptible | Resistant | Resistant | Resistant | Susceptible | Susceptible | Resistant | Resistant | - | - | - | - | - | - | - |
| RResHT-26 | Resistant | Susceptible | Susceptible | Resistant | - | Resistant | Susceptible | Resistant | Resistant | Resistant | - | - | - | - | - | Susceptible | - |
| RResHT-27 | Resistant | Susceptible | Susceptible | Resistant | - | Resistant | Susceptible | Resistant | Resistant | Resistant | - | - | - | - | - | - | - |
| RResHT-28 | Resistant | Susceptible | Susceptible | Resistant | Resistant | Resistant | Susceptible | Resistant | Resistant | Resistant | - | - | - | - | - | - | - |
| RResHT-29 | Resistant | Susceptible | Susceptible | Resistant | Resistant | Resistant | Resistant | Resistant | Resistant | Resistant | Resistant | Resistant | Resistant | Resistant | Susceptible | Resistant | Resistant |
| RResHT-30 | Resistant | Susceptible | Susceptible | Resistant | Resistant | Resistant | Resistant | - | - | Resistant | Susceptible | Susceptible | Susceptible | - | Resistant | Resistant | - |
| RResHT-31 | Susceptible | Susceptible | Susceptible | Resistant | - | - | Resistant | - | - | Resistant | Susceptible | Resistant | Susceptible | - | - | - | - |
| RResHT-32 | - | Susceptible | Susceptible | - | - | - | - | - | - | Resistant | - | - | - | - | - | - | - |
| RResHT-33 | - | Susceptible | Susceptible | Resistant | - | - | Susceptible | - | - | Resistant | - | - | - | - | - | - | - |
| RResHT-34 | Susceptible | Resistant | Resistant | Resistant | Resistant | Resistant | Resistant | Susceptible | Resistant | Resistant | Resistant | Resistant | Resistant | Susceptible | Resistant | Resistant | Resistant |
| RResHT-35 | - | Susceptible | Susceptible | Resistant | Resistant | Resistant | Susceptible | - | - | Resistant | - | - | - | - | - | - | - |
| RResHT-36 | - | Susceptible | Susceptible | Resistant | - | Resistant | Susceptible | - | - | Resistant | - | - | - | - | - | Resistant | - |
| RResHT-37 | Resistant | Resistant | Resistant | Resistant | Resistant | Resistant | Susceptible | Susceptible | Resistant | Resistant | Resistant | Resistant | Resistant | Susceptible | Susceptible | Resistant | Resistant |
| RResHT-38 | Susceptible | Resistant | Resistant | Resistant | Resistant | Resistant | Resistant | Resistant | Resistant | Resistant | Resistant | Resistant | Resistant | Resistant | Susceptible | Resistant | Resistant |
| RResHT-39 | Resistant | Resistant | Resistant | Susceptible | Resistant | Resistant | Susceptible | Susceptible | Resistant | Resistant | Resistant | Susceptible | Resistant | Susceptible | Resistant | Resistant | Resistant |
| RResHT-41 | - | Susceptible | Susceptible | Resistant | - | Resistant | Susceptible | - | - | Resistant | - | - | - | - | - | Resistant | - |
| RResHT-42 | - | Susceptible | Susceptible | Resistant | - | Resistant | Susceptible | - | - | - | - | - | - | - | - | Resistant | - |
| RResHT-44 | Susceptible | Resistant | Susceptible | - | Resistant | Susceptible | Susceptible | Susceptible | - | Susceptible | Resistant | Resistant | Resistant | Susceptible | Susceptible | Susceptible | Resistant |
| RResHT-45 | Susceptible | Resistant | Susceptible | Susceptible | Resistant | Resistant | Resistant | Resistant | Susceptible | Resistant | Susceptible | Resistant | Susceptible | Susceptible | Susceptible | Susceptible | Resistant |
| RResHT-46 | - | Susceptible | - | - | - | - | - | - | Resistant | - | - | - | - | - | - | - | - |
| RResHT-47 | - | Susceptible | Susceptible | Resistant | - | Resistant | Susceptible | - | Resistant | - | - | - | - | - | - | Resistant | - |
| RResHT-48 | - | Resistant | Susceptible | Resistant | - | Resistant | - | - | Resistant | Resistant | - | - | - | - | - | - | - |
| RResHT-49 | - | Susceptible | - | - | - | - | - | - | Resistant | Resistant | - | - | - | - | - | - | - |
| RResHT-50 | - | Susceptible | Susceptible | Resistant | - | Resistant | Susceptible | - | - | - | - | - | - | - | - | Resistant | - |
| RResHT-51 | Susceptible | Susceptible | Susceptible | Resistant | Resistant | Resistant | Resistant | Susceptible | Resistant | Resistant | Susceptible | Susceptible | Susceptible | Susceptible | Resistant | Susceptible | Resistant |
| RResHT-52 | Susceptible | Susceptible | Resistant | Susceptible | Resistant | Resistant | Resistant | Susceptible | Resistant | Resistant | Resistant | Resistant | Susceptible | Susceptible | Susceptible | Resistant | Resistant |
| RResHT-53 | Susceptible | Susceptible | Susceptible | Susceptible | Resistant | Resistant | Resistant | - | Susceptible | Resistant | Resistant | Resistant | Susceptible | Susceptible | Susceptible | Susceptible | Resistant |
| RResHT-54 | - | Susceptible | Susceptible | Resistant | - | - | - | - | Resistant | Resistant | - | - | - | - | - | - | - |
| RResHT-55 | - | Susceptible | Susceptible | Resistant | Resistant | Resistant | Susceptible | - | - | - | - | - | - | - | - | - | - |
| RResHT-56 | - | Susceptible | - | - | - | - | - | - | - | - | - | - | - | - | - | - | - |
| RResHT-57 | - | Susceptible | - | - | - | - | - | - | Resistant | - | - | - | - | - | - | - | - |
| RResHT-58 | - | Susceptible | - | - | - | - | - | - | Resistant | Resistant | - | - | - | - | - | - | - |
| RResHT-60 | Resistant | Susceptible | Resistant | Resistant | Resistant | Resistant | Resistant | Resistant | Resistant | Resistant | Resistant | Resistant | Resistant | Resistant | Resistant | Resistant | Resistant |
| RResHT-62 | Resistant | Resistant | Resistant | Resistant | Resistant | Resistant | Resistant | Resistant | Resistant | Resistant | Resistant | Resistant | Resistant | Resistant | Resistant | Resistant | Resistant |
| RResHT-63 | Resistant | Susceptible | Resistant | Resistant | Resistant | Resistant | Resistant | Resistant | Resistant | Resistant | Resistant | Resistant | Resistant | Resistant | Resistant | Resistant | Resistant |
| RResHT-64 | Resistant | - | - | Resistant | Resistant | - | Susceptible | - | - | Resistant | Resistant | Resistant | Resistant | - | Resistant | Resistant | - |
| RResHT-65 | - | - | - | Resistant | - | - | Susceptible | - | - | Resistant | Resistant | Susceptible | Resistant | - | - | - | - |
| RResHT-66 | Resistant | Susceptible | Resistant | Resistant | Resistant | Resistant | Resistant | Resistant | Resistant | Resistant | Resistant | Resistant | Resistant | - | Resistant | Resistant | Resistant |
| RResHT-67 | Resistant | Susceptible | Resistant | Resistant | - | Resistant | Resistant | Resistant | Resistant | Resistant | Resistant | Resistant | Resistant | - | Resistant | Resistant | Resistant |
| RResHT-68 | - | Resistant | Resistant | - | - | - | - | - | - | - | - | - | Resistant | - | - | - | Resistant |
| RResHT-69 | Resistant | Resistant | Susceptible | Resistant | Resistant | Resistant | Resistant | Resistant | Resistant | Resistant | Resistant | Resistant | Susceptible | Resistant | Resistant | Resistant | Resistant |
| RResHT-70 | - | Susceptible | Susceptible | - | Resistant | Resistant | Susceptible | - | Resistant | Susceptible | Resistant | Susceptible | Susceptible | - | Resistant | Resistant | Resistant |
| RResHT-71 | - | Susceptible | Resistant | Resistant | Resistant | Susceptible | Resistant | - | Resistant | Resistant | Resistant | - | Susceptible | Resistant | Resistant | - | Resistant |
| RResHT-72 | Resistant | Susceptible | Susceptible | Resistant | - | Resistant | Susceptible | Susceptible | Resistant | Susceptible | - | - | - | - | - | Resistant | - |
| RResHT-73 | Susceptible | Resistant | Susceptible | Resistant | Resistant | Resistant | Susceptible | Resistant | Resistant | Resistant | Resistant | Resistant | Susceptible | Resistant | Resistant | Resistant | Resistant |
| RResHT-74 | Susceptible | Susceptible | Susceptible | Resistant | - | Resistant | Susceptible | Resistant | Susceptible | Resistant | - | - | - | - | - | Susceptible | - |
| RResHT-75 | Resistant | Susceptible | Susceptible | - | - | - | - | Susceptible | Resistant | Susceptible | - | - | - | - | - | - | - |
| RResHT-76 | Susceptible | Susceptible | Susceptible | - | Resistant | Resistant | - | Susceptible | Resistant | Resistant | - | - | Susceptible | - | - | - | Resistant |
| RResHT-78 | Resistant | Susceptible | Susceptible | - | - | - | - | Susceptible | Susceptible | Resistant | - | - | - | - | - | - | - |
| RResHT-79 | Resistant | Susceptible | Susceptible | - | - | - | - | Susceptible | Susceptible | Resistant | - | - | - | - | - | - | - |
| RResHT-80 | Susceptible | Susceptible | Susceptible | - | - | - | - | Susceptible | Resistant | Resistant | - | - | - | - | - | - | - |
| RResHT-82 | Resistant | Susceptible | Susceptible | - | - | - | - | Susceptible | Resistant | Resistant | - | - | - | - | - | - | - |
| RResHT-83 | Resistant | Susceptible | Susceptible | - | - | - | - | Susceptible | Resistant | Susceptible | - | - | - | - | - | - | - |
| RResHT-84 | Susceptible | Susceptible | Susceptible | Resistant | Resistant | Resistant | Resistant | Susceptible | Resistant | Resistant | - | - | - | - | - | - | - |
| RResHT-85 | Susceptible | Susceptible | Susceptible | Resistant | - | Resistant | Susceptible | Susceptible | Resistant | Resistant | - | - | - | - | - | - | - |
| RResHT-86 | Susceptible | Susceptible | Susceptible | - | - | - | - | Susceptible | Resistant | Resistant | - | - | - | - | - | - | - |
| RResHT-87 | Susceptible | Susceptible | Susceptible | - | - | - | - | Susceptible | Resistant | Resistant | - | - | - | - | - | - | - |
| RResHT-91 | - | Susceptible | Susceptible | Resistant | - | - | Susceptible | - | Resistant | Resistant | - | - | - | - | - | - | - |
| RResHT-92 | - | Susceptible | Susceptible | Resistant | - | - | Susceptible | - | - | Resistant | - | - | - | - | - | - | - |
| RResHT-93 | - | Susceptible | Susceptible | - | - | - | - | - | - | Resistant | - | - | - | - | - | - | - |
| RResHT-94 | - | Susceptible | - | - | - | - | - | - | - | Resistant | - | - | - | - | - | - | - |
| RResHT-95 | Susceptible | Susceptible | Susceptible | Resistant | Resistant | Resistant | Resistant | Susceptible | Resistant | Resistant | Susceptible | Susceptible | Susceptible | Susceptible | - | Resistant | Resistant |
| RResHT-96 | Susceptible | Susceptible | Susceptible | Resistant | Resistant | Resistant | Resistant | Resistant | Resistant | Resistant | Susceptible | Susceptible | Susceptible | Susceptible | - | Susceptible | Resistant |
| RResHT-97 | Susceptible | Susceptible | Susceptible | Resistant | Resistant | Resistant | Resistant | Resistant | Resistant | Resistant | Resistant | Susceptible | Susceptible | - | Resistant | Susceptible | Resistant |
| RResHT-98 | Susceptible | Susceptible | Susceptible | Resistant | Resistant | Resistant | Susceptible | Susceptible | Resistant | Resistant | Resistant | Susceptible | Susceptible | Susceptible | Resistant | Resistant | Resistant |
| RResHT-99 | Susceptible | Susceptible | Susceptible | Resistant | Resistant | Resistant | Susceptible | Resistant | Resistant | Resistant | Resistant | Resistant | Susceptible | Resistant | Resistant | Resistant | Resistant |
| RResHT-100 | Susceptible | Resistant | Susceptible | Resistant | - | Resistant | Resistant | Susceptible | Resistant | Resistant | Resistant | Susceptible | Susceptible | Resistant | - | Resistant | Resistant |
| RResHT-101 | - | Susceptible | Susceptible | - | - | - | Susceptible | - | Resistant | - | Susceptible | Resistant | Resistant | Resistant | Resistant | - | Resistant |
| RResHT-102 | - | - | Susceptible | - | Resistant | Resistant | - | - | - | - | - | - | Susceptible | - | - | - | Resistant |

**Supplementary Table 3:** *Zymoseptoria tritici* isolates used in this study together with their collection dates and the wheat varieties from which they were sampled.

| ***Z. tritici* isolate** | **Year collected** | **Wheat variety from which isolate was sourced** |
| --- | --- | --- |
| RResHT-1 | 2015 | Consort |
| RResHT-2 | 2015 | Gallant |
| RResHT-3 | 2015 | KWS Cashel |
| RResHT-4 | 2015 | KWS Cashel |
| RResHT-5 | 2015 | KWS Cashel |
| RResHT-6 | 2016 | Consort |
| RResHT-7 | 2016 | Consort |
| RResHT-8 | 2016 | Cougar |
| RResHT-9 | 2016 | Consort |
| RResHT-10 | 2015 | Gallant |
| RResHT-11 | 2015 | Zulu |
| RResHT-12 | 2015 | Cougar |
| RResHT-13 | 2015 | Horatio |
| RResHT-14 | 2015 | Cougar |
| RResHT-15 | 2015 | Cougar |
| RResHT-16 | 2016 | Consort |
| RResHT-17 | 2015 | Cougar |
| RResHT-18 | 2015 | KWS Santiago |
| RResHT-19 | 2015 | JB Diego |
| RResHT-20 | 2015 | Cougar |
| RResHT-21 | 2015 | Cougar |
| RResHT-22 | 2015 | KWS Cashel |
| RResHT-23 | 2016 | Consort |
| RResHT-24 | 2016 | Consort |
| RResHT-25 | 2016 | Consort |
| RResHT-26 | 2015 | Cougar |
| RResHT-27 | 2015 | Gallant |
| RResHT-28 | 2015 | Cougar |
| RResHT-29 | 2016 | Cordiale |
| RResHT-30 | 2016 | Cordiale |
| RResHT-31 | 2016 | Cordiale |
| RResHT-32 | 2016 | Cordiale |
| RResHT-33 | 2016 | Cordiale |
| RResHT-34 | 2016 | Cordiale |
| RResHT-35 | 2016 | Cordiale |
| RResHT-36 | 2016 | Cordiale |
| RResHT-37 | 2016 | Cordiale |
| RResHT-38 | 2016 | Cordiale |
| RResHT-39 | 2016 | Cordiale |
| RResHT-40 | 2016 | Dickens |
| RResHT-41 | 2016 | Dickens |
| RResHT-42 | 2016 | Dickens |
| RResHT-43 | 2016 | Dickens |
| RResHT-44 | 2016 | Dickens |
| RResHT-45 | 2016 | KWS Santiago |
| RResHT-46 | 2016 | KWS Santiago |
| RResHT-47 | 2016 | Alchemy |
| RResHT-48 | 2016 | Cordiale |
| RResHT-49 | 2015 | KWS Cashel |
| RResHT-50 | 2016 | Dickens |
| RResHT-51 | 2016 | Dickens |
| RResHT-52 | 2016 | Dickens |
| RResHT-53 | 2015 | KWS Cashel |
| RResHT-54 | 2016 | Dickens |
| RResHT-55 | 2016 | Dickens |
| RResHT-56 | 2016 | Dickens |
| RResHT-57 | 2016 | Dickens |
| RResHT-58 | 2016 | Dickens |
| RResHT-59 | 2016 | Dickens |
| RResHT-60 | 2016 | Dickens |
| RResHT-61 | 2016 | Dickens |
| RResHT-62 | 2016 | Dickens |
| RResHT-63 | 2016 | Dickens |
| RResHT-64 | 2015 | KWS Cashel |
| RResHT-65 | 2015 | KWS Cashel |
| RResHT-66 | 2015 | KWS Cashel |
| RResHT-67 | 2015 | KWS Cashel |
| RResHT-68 | 2015 | KWS Cashel |
| RResHT-69 | 2015 | KWS Cashel |
| RResHT-70 | 2015 | KWS Cashel |
| RResHT-71 | 2015 | KWS Cashel |
| RResHT-72 | 2015 | Dickens |
| RResHT-73 | 2016 | Reflection |
| RResHT-74 | 2017 | Siskin |
| RResHT-75 | 2017 | Siskin |
| RResHT-76 | 2017 | Siskin |
| RResHT-77 | 2017 | Siskin |
| RResHT-78 | 2017 | Siskin |
| RResHT-79 | 2017 | Siskin |
| RResHT-80 | 2017 | Siskin |
| RResHT-81 | 2017 | Siskin |
| RResHT-82 | 2017 | Siskin |
| RResHT-83 | 2017 | Siskin |
| RResHT-84 | 2017 | Siskin |
| RResHT-85 | 2015 | Cougar |
| RResHT-86 | 2015 | Cougar |
| RResHT-87 | 2015 | Cougar |
| RResHT-88 | 2015 | Cougar |
| RResHT-89 | 2015 | Cougar |
| RResHT-90 | 2016 | Evolution |
| RResHT-91 | 2016 | Evolution |
| RResHT-92 | 2016 | Evolution |
| RResHT-93 | 2016 | JB Diego |
| RResHT-94 | 2016 | Zulu |
| RResHT-95 | 2016 | Dickens |
| RResHT-96 | 2016 | Dickens |
| RResHT-97 | 2016 | Consort |
| RResHT-98 | 2016 | Reflection |
| RResHT-99 | 2016 | Reflection |
| RResHT-100 | 2016 | Reflection |
| RResHT-101 | 2016 | Reflection |
| RResHT-102 | 2017 | Siskin |
| RResHT-103 | 2017 | Siskin |
| RResHT-104 | 2016 | Evolution |

**Supplementary Table 4:** Table showing the number of *Z. tritici* isolates in the test set originally isolated from each wheat variety in the field (along with the standard control isolate IPO 323).

| Wheat variety from which isolate was sourced | No. isolates |
| --- | --- |
| Alchemy | 1 |
| Consort | 9 |
| Cordiale | 12 |
| Cougar | 14 |
| Dickens | 21 |
| Evolution | 4 |
| Gallant | 3 |
| Horatio | 1 |
| JB Diego | 2 |
| KWS Cashel | 14 |
| KWS Santiago | 3 |
| Reflection | 5 |
| Siskin | 13 |
| Zulu | 2 |
